# Immune Clearance of Attenuated Rabies Virus Results in Neuronal Survival with Altered Gene Expression

**DOI:** 10.1371/journal.ppat.1002971

**Published:** 2012-10-11

**Authors:** Emily A. Gomme, Christoph Wirblich, Sankar Addya, Glenn F. Rall, Matthias J. Schnell

**Affiliations:** 1 Department of Microbiology and Immunology, Jefferson Medical College, Thomas Jefferson University, Philadelphia, Pennsylvania, United States of America; 2 Kimmel Cancer Center, Jefferson Medical College, Thomas Jefferson University, Philadelphia, Pennsylvania, United States of America; 3 Fox Chase Cancer Center, Philadelphia, Pennsylvania, United States of America; 4 Jefferson Vaccine Center, Jefferson Medical College, Thomas Jefferson University, Philadelphia, Pennsylvania, United States of America; University of Iowa, United States of America

## Abstract

Rabies virus (RABV) is a highly neurotropic pathogen that typically leads to mortality of infected animals and humans. The precise etiology of rabies neuropathogenesis is unknown, though it is hypothesized to be due either to neuronal death or dysfunction. Analysis of human brains post-mortem reveals surprisingly little tissue damage and neuropathology considering the dramatic clinical symptomology, supporting the neuronal dysfunction model. However, whether or not neurons survive infection and clearance and, provided they do, whether they are functionally restored to their pre-infection phenotype has not been determined *in vivo* for RABV, or any neurotropic virus. This is due, in part, to the absence of a permanent “mark” on once-infected cells that allow their identification long after viral clearance. Our approach to study the survival and integrity of RABV-infected neurons was to infect Cre reporter mice with recombinant RABV expressing Cre-recombinase (RABV-Cre) to switch neurons constitutively expressing tdTomato (red) to expression of a Cre-inducible EGFP (green), permanently marking neurons that had been infected *in vivo*. We used fluorescence microscopy and quantitative real-time PCR to measure the survival of neurons after viral clearance; we found that the vast majority of RABV-infected neurons survive both infection and immunological clearance. We were able to isolate these previously infected neurons by flow cytometry and assay their gene expression profiles compared to uninfected cells. We observed transcriptional changes in these “cured” neurons, predictive of decreased neurite growth and dysregulated microtubule dynamics. This suggests that viral clearance, though allowing for survival of neurons, may not restore them to their pre-infection functionality. Our data provide a proof-of-principle foundation to re-evaluate the etiology of human central nervous system diseases of unknown etiology: viruses may trigger permanent neuronal damage that can persist or progress in the absence of sustained viral antigen.

## Author Summary

Rabies is an ancient and fatal neurological disease of animals and humans, caused by infection of the central nervous system (CNS) with *Rabies virus* (RABV). It is estimated that nearly 55,000 human RABV fatalities occur each year, though this number is likely much higher due to unreported exposures or failure of diagnosis. No treatment has been identified to cure disease after onset of symptoms. Neurovirologists still do not know the cause of rabies' dramatic symptoms and fatality, though it is thought to be due to neuronal loss or dysfunction. Here, we use a novel approach to permanently and genetically tag infected cells so that they can be identified after the infection has been cleared. This allowed us to define neuronal survival time following infection, and to assess neuronal function through gene expression analysis. We found that RABV infection does not lead to loss of neurons, but rather induces a permanent change in gene expression that may be related to the ability of RABV to cause permanent CNS disease. Our study provides evidence that viral infection of the brain can initiate long-term changes that may have consequences for nervous system health, even after the virus has been cleared from the CNS.

## Introduction

Rabies is a fatal neurological disease of animals and humans for which there is no treatment once symptoms develop. The disease is caused by infection of the central nervous system (CNS) with the single-stranded RNA virus, *Rabies virus* (RABV). Infection results in dramatic neurological symptoms—aggression, hyperactivity, muscle weakness, paralysis, coma—invariably leading to fatality. The precise etiology of rabies pathogenesis is unknown and hypothesized to be either neuronal death or dysfunction. However, whether infected neurons can survive infection and the resultant immune response is unknown. Moreover, if these neurons survive, whether they are functionally restored to their pre-infection competence has not been determined *in vivo* for RABV, or for any neurotropic virus.

Analysis of human brains post-mortem reveals surprisingly little tissue damage and neuropathology, considering the dramatic clinical symptomology [1], [2]. As seen for other viral infections both RABV replication and resultant anti-viral immune responses are believed to be non-cytolytic; the latter mediated by cytokines, including type I interferons, and neutralizing antibodies [3]–[7]. Acute infection induces global upregulation of proinflammatory and innate immunity genes, including IL-6, TNF-α, type I interferons, complement cascade genes, and toll-like receptors within the brain [8]–[10]. Though there is some evidence that infection induces morphologic changes in infected neurons [11], [12], there is a distinct lack of overt histopathological changes indicative of apoptosis or necrosis [1], [2]. Attempts to recapitulate this *ex vivo* have been difficult; some viral strains induce neuronal apoptosis in tissue culture, while others do not [12]–[18]. This demonstrates the importance of studying neuronal cell fate in an animal model.

An alternative hypothesis is that neuronal dysfunction, rather than cell death, is responsible for the clinical features and fatal outcome in rabies. Neurological abnormalities are obvious, but studies in experimental rabies have revealed other phenomena, including disappearance of rapid eye movement (REM) sleep and initiation of facial twitching, called myoclonus, prior to development of classic symptoms. It was also found that brain electrical activity terminated about 30 minutes before cardiac arrest, indicating that cerebral death precedes organ failure [19], [20]. This correlates with functional deficiencies observed during acute experimental RABV infection, including altered expression of proteins involved in synapse communication and ion homeostasis [21], as well as neuronal depolarization and decreased neurotransmitter binding [22]–[26].

A challenge in studying the longevity of infected neurons is identifying and isolating them after resolution of the acute viral infection. In the absence of a permanent “mark” on once-infected cells, it is impossible to decisively answer the question as to whether infected cells survive and regain their pre-infection functionality. Instead, more general metrics of CNS health have been used, including histopathological assessments, absence of cell death (via assays with known limitations *in vivo*, such as TUNEL staining), and *in vitro* studies using neuroblastoma cells or primary neurons that may not faithfully recapitulate the biology of an infected neuron *in vivo*. Furthermore, control of viral replication by host immune responses in immunocompetent animal models may limit the infection to only a few cells, and apoptotic loss of these few cells may not be readily detected by most methods.

Here we present a novel approach to study neuronal cell fate after RABV infection. We infected Cre reporter mice with a sub-lethal dose of recombinant RABV expressing Cre-recombinase (RABV-Cre) to switch neurons constitutively expressing tdTomato (red) to expression of a Cre-inducible EGFP (green), permanently marking neurons that had been infected *in vivo*. This model allowed us to monitor neuronal survival after infection and to isolate neurons that resolved infection to characterize gene expression profiles relative to uninfected neurons. Our results support the notion that the majority of neurons survive infection, but remain impaired; this may account for the CNS disease caused by this neuropathogen.

## Results

### Recovery and characterization of Cre-expressing RABV (RABV-Cre)

**Figure 1:**
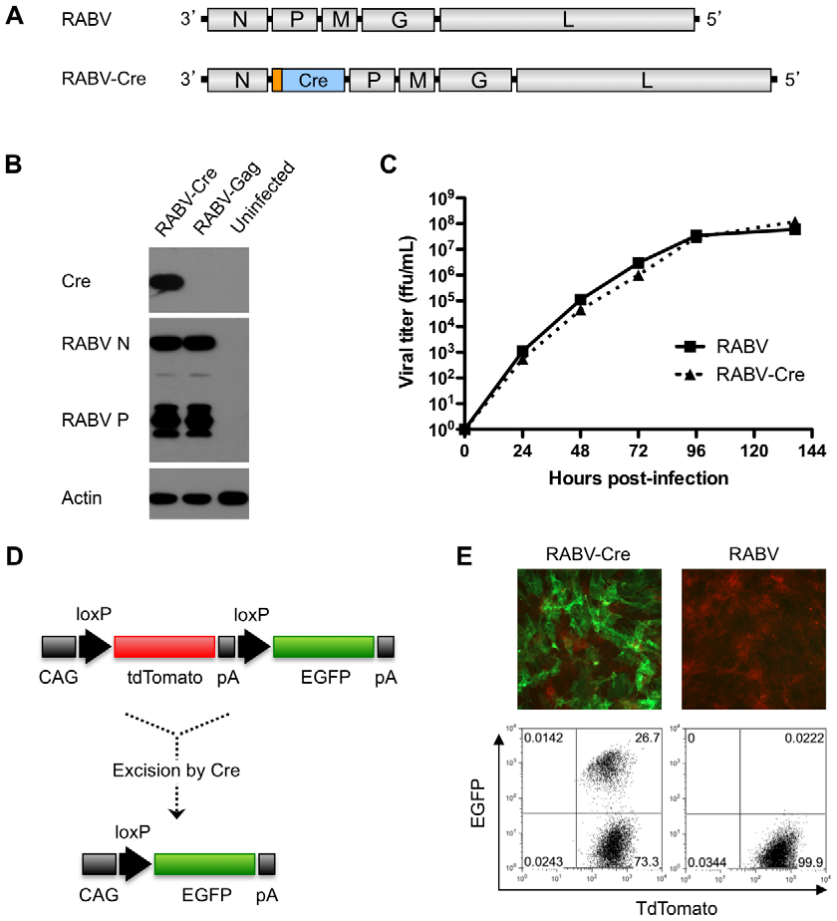
Characterization of Cre-expressing RABV. A) Genome of rabies virus (RABV) and the recombinant RABV expressing Enterobacteria phage P1 Cre recombinase (RABV-Cre) with a 5′ nuclear localization signal (shown in orange). B) Cre expression was confirmed by western blot analysis. Neuroblastoma cells were infected with either RABV-Cre, RABV expressing HIV-1 Gag (RABV-Gag), or mock infected (uninfected). Proteins were separated by sodium dodecyl sulfate-polyacrylamide gel electrophoresis and subjected to Western blotting with antibodies specific for Cre, RABV P or N, and actin. C) Viral growth kinetics were evaluated by multi-step growth curve assay in which BSR cells were infected at MOI 0.01 with either RABV or RABV-Cre, and viral titers determined from samples taken at the indicated time points post-infection. D) A schematic depicting the Cre-specific expression cassette in the Cre reporter mouse. In the absence of Cre, the chicken β-actin core promoter with a CMV enhancer (CAG) drives constitutive expression of membrane-targeted tandem dimer tomato (tdTomato) expression; EGFP is not expressed. After Cre-mediated excision of the tdTomato gene at the loxP sites, membrane-targeted enhanced green fluorescent protein (EGFP) is expressed. pA denotes polyadenylation sites. E) Cre functionality was evaluated *in vitro* by infecting primary fibroblasts isolated and cultured from Cre reporter mice for 96 hours with either RABV-Cre or RABV at MOI 20. EGFP and tdTomato labeling were detected by fluorescence microscopy (top) and flow cytometry (bottom) (using FITC and PE channels, respectively).

In order to identify and isolate cells (specifically, neurons) after resolution of a viral infection, we adopted a double-fluorescent Cre reporter mouse model [27]. These Cre reporter mice constitutively and ubiquitously express membrane-targeted tandem dimer Tomato (tdTomato); upon exposure to Cre recombinase, the tdTomato gene is deleted, and membrane-targeted EGFP is induced (Figure 1D). We generated a recombinant rabies virus (RABV-Cre) expressing Cre-recombinase (Figure 1A) [28], [29], which, in combination with the Cre reporter mouse, provided a model to permanently change the color of an infected cell from red to green, even after the virus was cleared. Cre was modified by the addition of a 5′ nuclear localization signal to promote high efficiency, *in vivo* recombination between *loxP* sites, and cloned into the an empty recombinant RABV backbone previously published as “BNSP” [30]. This recombinant virus is based on the SAD B19 RABV vaccine strain, which is moderately pathogenic after intracranial inoculation [30], but known to infect neurons efficiently [31]. Of note, to study long-term effects of RABV infection on neurons, it was imperative to choose a viral strain that efficiently infected the brain but that resulted in full recovery of the host: our previous work indicated that doses of this strain could be delivered that resulted in extensive neuronal infection, a robust antiviral response, and no mortality [30].

RABV-Cre was recovered by standard methods [29] and the fitness of the recombinant RABV, as well as the expression of the inserted Cre gene, was evaluated *ex vivo*. BSR fibroblasts were infected with RABV or RABV-Cre and analyzed by western blot for protein expression (Figure 1B) as well as one-step growth curves for viral replication (Figure 1C). These assays demonstrated that insertion of Cre between the RABV nucleoprotein (N) and phosphoprotein (P) genes had no effect on the rate of viral replication, or relative viral gene expression (Figure 1B and C). To analyze the functionality of the virus-encoded Cre, primary mouse fibroblasts harvested from Cre reporter mice were infected with RABV or RABV-Cre. Functional expression of Cre was indicated by EGFP expression in Cre reporter mouse fibroblasts after infection with RABV-Cre but not after infection with wildtype RABV (Figure 1D and E). Of note, these cells are double-labeled with EGFP and tdTomato; this delayed loss of tdTomato is likely due to a long protein half-life. Published data shows loss of tdTomato upwards of 9 days post-excision [27], though this may vary from tissue to tissue.

#### RABV infection of brain neurons in Cre reporter mouse are ‘permanently marked’ by constitutive EGFP expression

**Figure 2:**
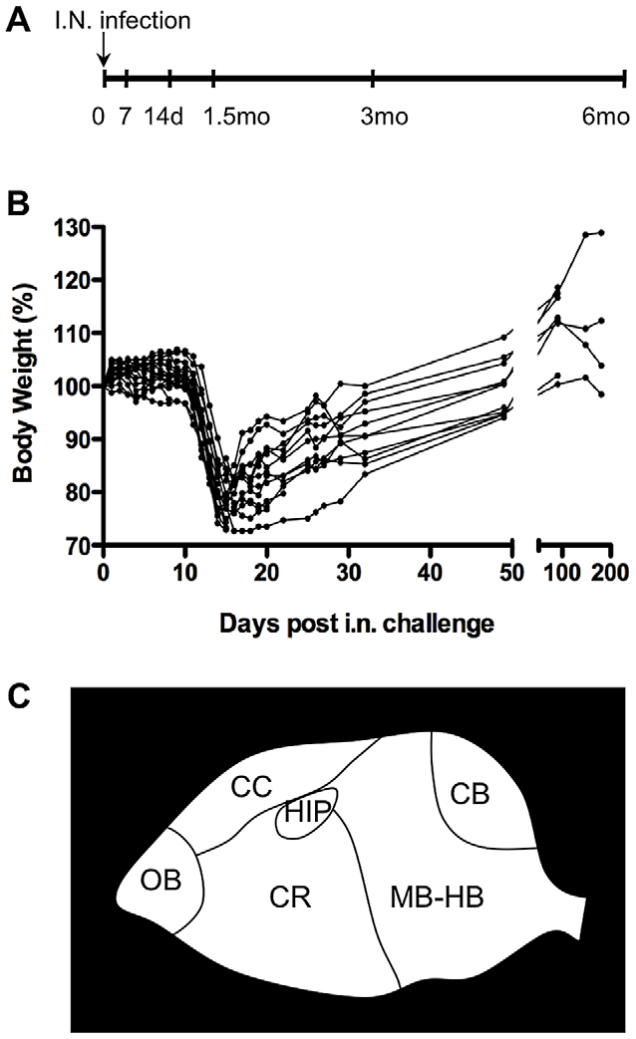
*In vivo* analysis of RABV-infected cells using Cre reporter mouse model. A) Timeline of mouse experiment. Cre reporter mice were infected intranasally (IN) with 10^5^ ffu RABV-Cre and sacrificed at the specified times. B) Weights of infected mice were monitored as a measure of disease throughout the experiment and demonstrate productive infection in all mice within this experiment. C) Brains collected at different time points post-infection were analyzed for the presence of EGFP-expressing cells in the following anatomical regions: olfactory bulb (OB), cerebral cortex (CC), cerebrum (CR), hippocampus (HIP), cerebellum (CB) and midbrain/hindbrain (MB-HB).

Cre reporter mice were infected intranasally (IN) with 10^5^ foci forming units (ffu) RABV-Cre (Figure 2A). This sub-lethal dose was specifically chosen to permit the study of neuronal survival and integrity at later time points post-infection. Infected mice became moribund by day 15 post-infection and developed classic signs of rabies—weight loss, fur ruffling, ataxia, hunched posture—though none displayed hind limb paralysis (Figure 2B). The lack of peripheral neuropathy, as seen before [32], is likely due to the route of administration, because IN-administered RABV directly invades the CNS without infecting the peripheral nervous system. 65% to 75% of the infected mice survived acute infection and regained body weight after day 15 (Figure 2B).

**Figure 3:**
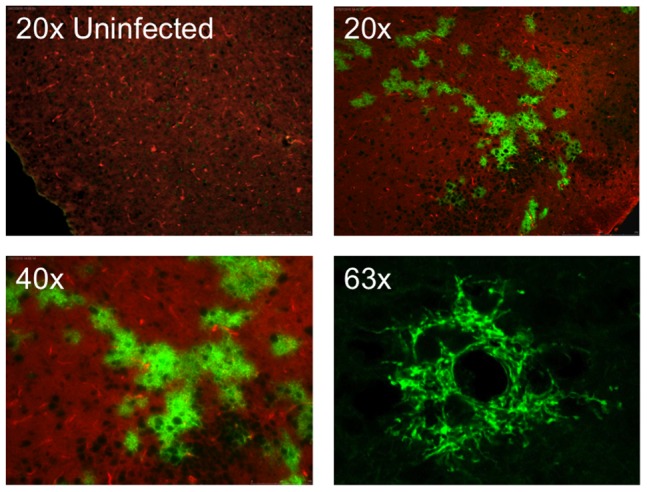
Visualization of EGFP-labeled cells from infected mouse brains during acute infection. Fifteen days post-infection, brains were collected, cryosectioned, and imaged without secondary staining on a standard fluorescent microscope (20× and 40× images) or confocal microscope (63× image). All images shown are from the olfactory bulb region.

**Figure 4:**
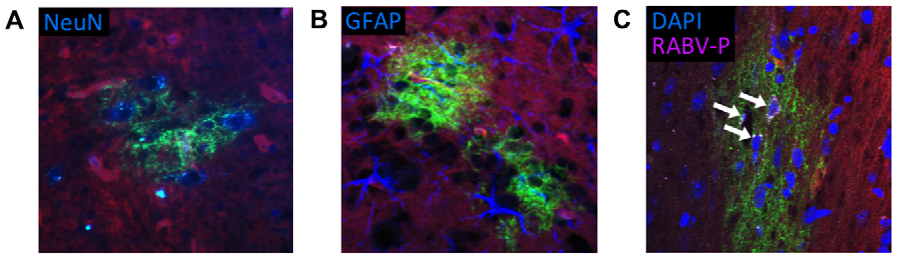
EGFP+ neurons are positive for RABV antigen. Brains were collected from Cre reporter mice fifteen days post-infection, cryosectioned, and EGFP+ regions compared to cell-specific labeling, A) NeuN (blue, neuronal nuclei antibody, 20× fluorescence imaging), B) GFAP (blue, astrocyte antibody, 40× confocal imaging), or C) RABV P antigen (purple) and DAPI nuclear stain (blue, 63× confocal imaging). White arrows in (C) indicate regions positive for RABV P.

Cre reporter mice were maintained up to 6 months post-infection with groups of 3–4 mice being sacrificed at the time points depicted in Figure 2A. At each time point, brains were collected from the mice for imaging and RNA analysis. We initially imaged brains collected from mice 15 days post-infection—a point when viral replication was still active [33], [34] and clinical signs of RABV peaked (Figure 2B). Only brain tissue from infected mice showed regions of EGFP expression amid non-transformed tdTomato-expressing brain tissue (Figure 3 and 4). Because the fluorophores are membrane-targeted, we were able to detect single-cell morphology including individual dendrites using confocal microscopy (Figure 3). To further characterize the EGFP+ regions after RABV infection, we stained for RABV P antigen and cell-specific markers for neurons (NeuN) and astrocytes (GFAP) (Figure 4). *In vivo*, RABV is known to replicate almost exclusively in neurons [35] and rarely in glial cells [2]. As shown in Figure 4C, we detected perinuclear RABV P amid clusters of infected neurons only within the EGFP+ regions.

#### Neurons survive RABV infection and viral clearance *in vivo*

**Figure 5:**
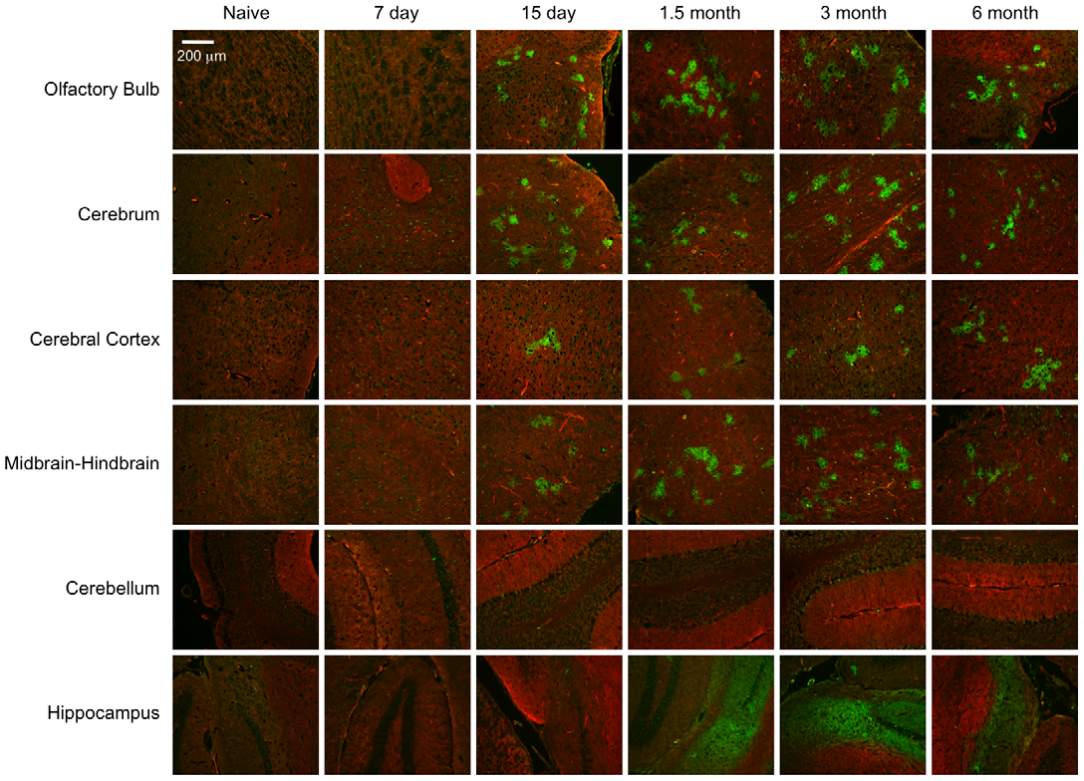
Neurons persist throughout the infected mouse brain long-after acute viral infection. The spread of the RABV infection was detected by identifying regions of EGFP fluorescence in different neuroanatomical regions (see Figure 2C) over a 6 month time course. Images are representative of 3–4 mice analyzed at each time point.

**Figure 6:**
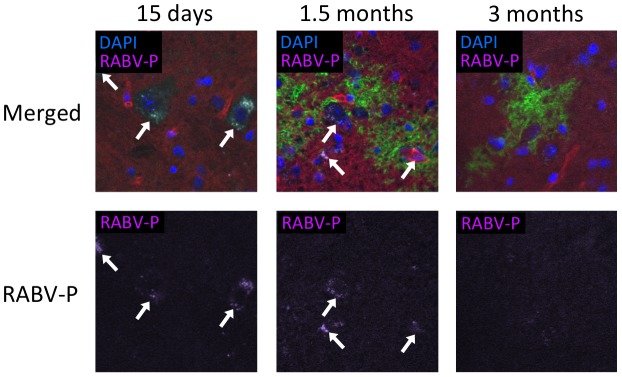
RABV antigen is undetectable by immunostaining 3 months after infection. Brains from infected mice were collected at the indicated time points and immunostained. The panels show cryosections stained for RABV P antigen (purple) and DAPI nuclear stain (blue; 63× confocal imaging). White arrows indicate regions positive for RABV P.

Having validated that the EGFP+ regions correspond to RABV-infected neurons, we examined the distribution and duration of EGFP foci over the course of 6 months during which RABV infection would have been cleared. Though rabies can be cleared as early as 18 days post-infection [33], [34], we detected EGFP+ foci out to 6 months post-infection (Figure 5). Single EGFP+ cells begin to appear as early as day 7 post-infection, but stable numbers of multi-nucleated foci were present in all areas evaluated in the brain parenchyma from day 15 through 6 months post-infection (Figures 5 and 6). Of note, the sizes of these foci were morphologically similar across this timeline. Only the hippocampus becomes EGFP+ later than the other regions, between 15 days and 1.5 months post-infection, but then remains unchanged through the rest of the time course. Moreover, the hippocampus is the most brightly labeled anatomical region of the brain. In contrast, the least prominent labeling was found in the cerebellum, though these levels of labeling are clearly higher than naïve cerebellar sections. This agrees with other reports that RABV is distributed throughout various regions of the brain, including cerebral cortex, hippocampus, and cerebellum [34], [36]. These neurons are either survivors of RABV infection, or newly-infected cells.

**Figure 7:**
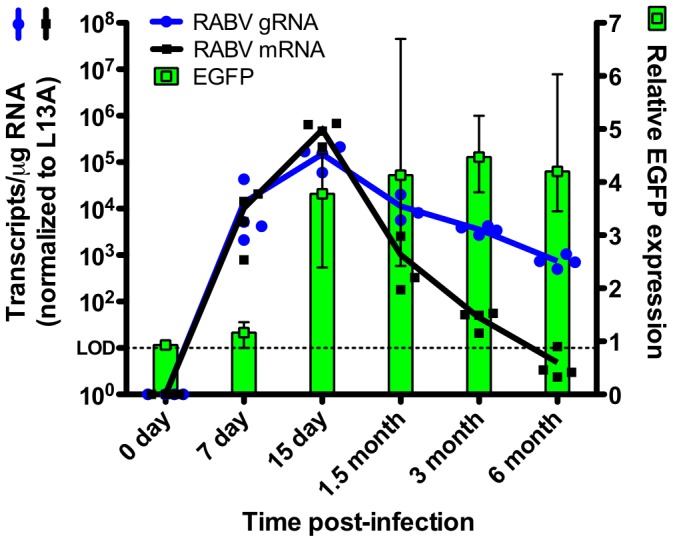
Neurons survive RABV infection and viral clearance. RNA was isolated from brains of mice at the indicated time points post-infection and assayed by real-time quantitative PCR for RABV genomic RNA (blue circles and line; left y-axis), RABV messenger RNA (black squares and line; left y-axis), and relative EGFP expression (green bars; right y-axis). Gene expression of all targets was normalized to the RPL13A (L13A) housekeeping gene. Data displayed was collected from 3 to 4 mice at each time point.

To elucidate whether EGFP+ foci observed at later time points are products of new infection or previously-infected neurons, we used quantitative real time PCR to compare levels of viral genome and messenger RNA to that of EGFP from the same brains used to image the tdTomato/EGFP in Figure 5. Similar to the immunofluorescence data, EGFP RNA peaks at day 15 post-infection and remains stably expressed through our last time point, 6 months post-infection (Figure 7). In contrast, active RABV infection, as measured by RABV N mRNA production, peaks at day 15 post-infection and decreases thereafter, with fewer than 100 copies of viral message detected by 3 months post-infection. This is supported by immunohistochemical detection of RABV P at these time points (Figure 6). While others have reported viral mRNA peaking at days 6–8 post-infection with near complete clearance by day 18 [33], our more sensitive quantification shows sustained viral persistence, particularly of the genome that is still present at approximately 1000 copies as late as 6 months post-infection (Figure 7). It has been suggested that viral genome persistence for negative-stranded RNA viruses is due to the stabilizing effects of the nucleoprotein which closely encapsidates the genome, and not necessarily to the production of new genomes. Because negative-stranded RNA viruses cannot replicate without the production of the RABV N, P, or L proteins, the lack of N mRNA clearly indicates the lack of active replication.

### Microarray analysis of RABV-infected neurons isolated by FACS 3 months after infection indicates gene dysregulation

**Figure 8:**
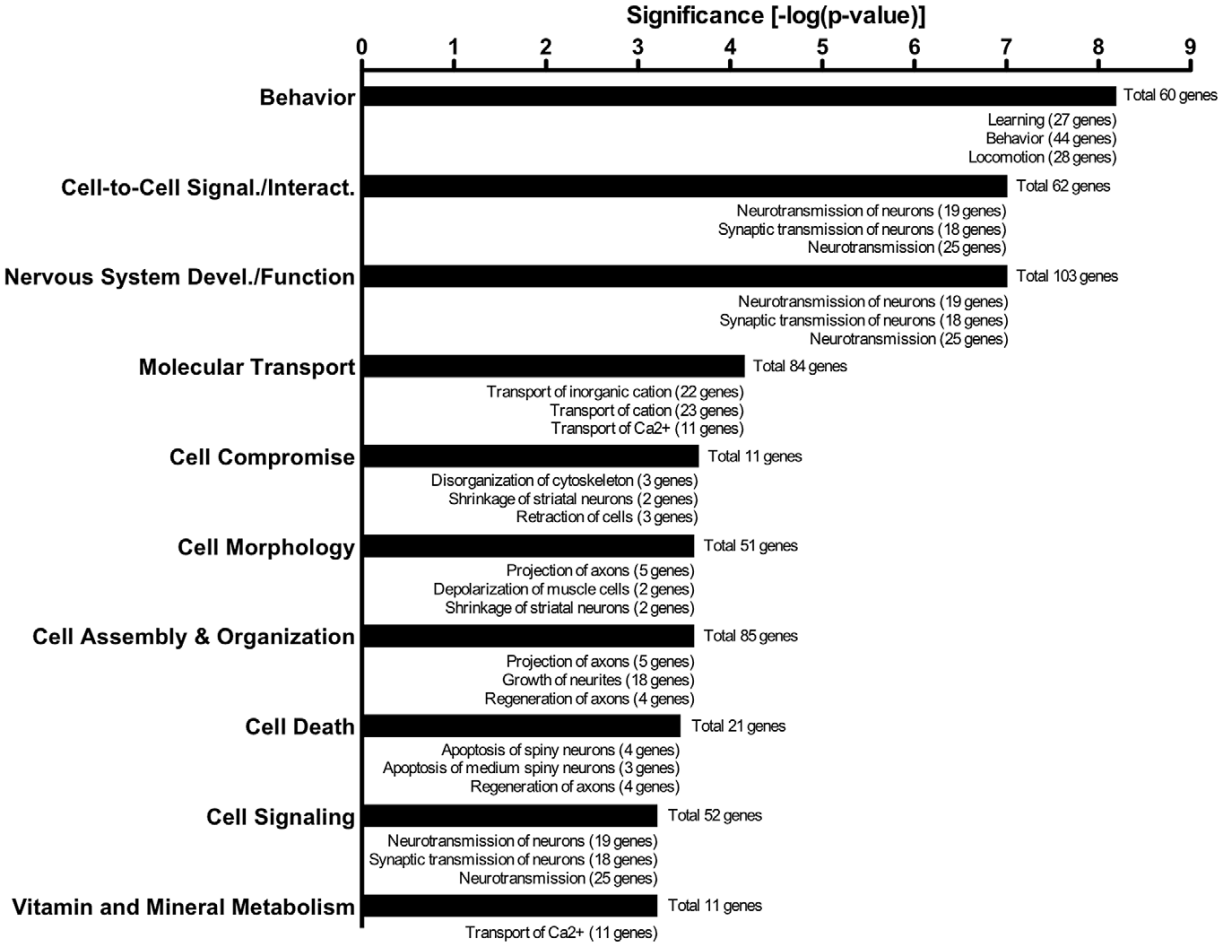
Microarray analysis of RABV-infected neurons isolated by FACS 3 months after infection indicates dysregulation of genes involved in nervous system function and cellular assembly. Cell suspensions prepared from whole mouse brains 3 months post-infection were sorted on a MoFlo cell sorter for EGFP+ (previously infected) and EGFP- (uninfected) cell populations. The 1248 transcripts differentially expressed between infected and uninfected cells (≥1.5 fold change, p<0.05) were analyzed by Ingenuity Pathway Analysis (IPA) to identify biological functions most significantly affected by the infection (significance predicted by p-value). Shown are the top ten most significant biological systems affected by the gene dysregulation, with the horizontal bars representing the negative log of their p-value (greatest significance at the top). Below each bar is the top three sub-categories affected by gene dysregulation in the respective categories. Each category/sub-category has the number of genes involved (up or down-regulated).

Neuronal survival following viral infection next prompted us to investigate the functional integrity of these “cured cells”. We used fluorescence activated cell sorting (FACS) to isolate the EGFP+ population (“infected”) and the tdTomato+ population (“uninfected”) from individual mice 3 months post-infection (Supplemental Figure S1), a time when viral transcription was undetectable (Figure 7). Of note, the endogenous fluorescence of these populations precluded the need for intracellular staining that compromises RNA integrity. Affymetrix Gene Microarray was used to measure transcriptional profiles of the infected and uninfected cells from two independent cell sorting events (n = 2 for each group). This analysis identified 1248 genes differentially expressed between the sorted groups (infected vs. uninfected, ≥1.5 fold change, p<0.05), 361 genes of which were down-regulated, 887 genes that were up-regulated. Gene expression levels ranged from 3.09-fold up-regulation to 2.72-fold down-regulation, and only 127 genes differed by more than 2-fold (Supplemental Table S1). Though the overall change in gene expression was modest, these transcriptional changes may be implicated in neuronal dysfunction. We saw no decrease in neuron-specific genes classically down-regulated in neurological disorders; in fact, there was up-regulation of neuronal receptors (glutamate receptor, GABA receptor), ion channels (potassium, sodium, and hydrogen), neurotransmitter transporter (Cacna1b), and synapse-specific genes (synaptotagmin I, synaptophysin) (Supplemental Table S2), strongly supporting our contention that these analyzed cells are of neuronal origin. However, it is not a given that the change in expression of “neuron-specific” genes is responsible for the dysfunction of the neuron. More likely it is the virus' interaction with ubiquitous cellular genes that induces functional changes within the neuron. For example, genes encoding proteins that play critical roles in ion homeostasis, exocytosis, or mitochondrial function have been shown to have a great effect on neuronal function – however these genes are commonly expressed in a large variety of cells. Ingenuity Pathway Analysis (IPA) was used to identify biological functions most significantly impacted; cell-to-cell signaling was the most significant molecular and cellular function impacted, whereas behavior and nervous system development/function were the two most significant physiological systems impacted (Figure 8). Analyzing the regulation of groups of related genes, IPA predicted decreased function (those with an absolute z-score value≥1.96) in the following areas: neurite growth/outgrowth, organization of cytoskeleton, organization of cytoplasm, and microtubule dynamics (genes involved in these functions are listed in Table 1).

**Table 1:** Dysregulated genes and predicted effect on cell functions using Ingenuity Pathway Analysis.

Functions Affected*	Predicted Effect on Function**	Gene Description	Gene Symbol	Transcripts Cluster ID	Fold Change
Neur	Increased	apurinic/apyrimidinic endonuclease 1; transmembrane protein 55b	APEX1	10414522	1.602
Cskel, Cplsm, Micr	Increased	BCL2-associated athanogene 4	BAG4	10577858	1.660
Neur	Affected	calcium channel, voltage-dependent, P/Q type, alpha 1A subunit	CACNA1A	10573348	1.893
Cskel, Cplsm, Micr	Increased	calpastatin	CAST	10410656	−1.646
Cskel, Cplsm, Micr	Decreased	cyclin-dependent kinase 5, regulatory subunit 1 (p35)	CDK5R1	10379482	2.168
Cskel, Cplsm, Micr	Increased	cyclin-dependent kinase inhibitor 1B	CDKN1B	10542317	1.741
Cskel, Cplsm, Micr	Decreased	CLIP associating protein 1	CLASP1	10349249	1.520
Neur	Increased	cannabinoid receptor 1 (brain)	CNR1	10503902	2.179
Cskel, Cplsm	Affected	contactin associated protein-like 1	CNTNAP1	10381272	1.835
Neur, Cskel, Cplsm, Micr	Decreased	cAMP responsive element binding protein 1	CREB1	10346943	−1.684
Cskel, Cplsm	Affected	colony stimulating factor 1 (macrophage)	CSF1	10501164	−1.679
Cskel, Cplsm, Micr	Affected	doublecortin-like kinase 1; predicted gene 9831	DCLK1	10498204	1.630
Neur	Decreased	delta/notch-like EGF-related receptor	DNER	10356177	1.786
Cplsm	Affected	endothelial PAS domain protein 1	EPAS1	10447317	−1.552
Neur	Affected	Eph receptor A4	EPHA4	10355893	1.925
Neur	Decreased	Fas apoptotic inhibitory molecule	FAIM	10588083	−1.847
Cskel, Cplsm, Micr	Decreased	fibroblast growth factor 1	FGF1	10458560	−2.180
Cskel, Cplsm	Affected	flightless I homolog (Drosophila)	FLII	10386551	−1.537
Neur, Cskel, Cplsm	Decreased	fibronectin 1	FN1	10355403	−1.637
Cskel, Cplsm, Micr	Decreased	tyrosine-protein kinase Fyn	FYN	10362596	−1.688
Cplsm	Affected	glucosidase, alpha, acid	GAA	10383088	1.698
Cskel, Cplsm, Micr	Decreased	histone deacetylase 6	HDAC6	10603387	1.521
Cplsm	Affected	hexosaminidase A	HEXA	10585874	2.105
Neur	Decreased	high mobility group box 1	HMGB1	10604663	−2.295
Cskel, Cplsm, Micr	Increased	huntingtin	HTT	10521261	1.909
Cskel, Cplsm, Micr	Affected	hydrocephalus inducing	HYDIN	10575380	−2.241
Cskel, Cplsm, Micr	Decreased	kinesin family member 5A	KIF5A	10373113	−1.671
Cplsm	Affected	kinesin family member 5B	KIF5B	10457273	−1.842
Cskel, Cplsm, Micr	Decreased	kinesin light chain 1	KLC1	10398727	−1.521
Cskel, Cplsm	Affected	keratin 17	KRT17	10391066	1.550
Neur	Decreased	leucine-rich repeat kinase 2	LRRK2	10426315	1.793
Cskel, Cplsm	Affected	lymphocyte specific 1	LSP1	10559207	−1.590
Cskel, Cplsm, Micr	Increased	mitogen-activated protein kinase 8	MAPK8	10418879	1.716
Cskel, Cplsm	Decreased	mannoside acetylglucosaminyltransferase 5	MGAT5	10349404	1.967
Cskel, Cplsm	Affected	metastasis suppressor 1	MTSS1	10428857	−1.716
Neur, Cskel, Cplsm, Micr	Decreased	myosin, heavy polypeptide 9, non-muscle	MYH9	10430201	−1.774
Cskel, Cplsm	Affected	myosin IF	MYO1F	10443980	−1.519
Cskel, Cplsm, Micr	Affected	NCK-associated protein 1	NCKAP1	10484318	1.721
Neur, Cskel, Cplsm, Micr	Decreased	neuropilin 1	NRP1	10576639	1.760
Cskel, Cplsm	Affected	piccolo (presynaptic cytomatrix protein)	PCLO	10519770	1.834
Neur	Decreased	phospholipase D1	PLD1	10491106	−1.677
Cskel, Cplsm, Micr	Decreased	prion protein	PRNP	10476314	1.607
Cskel, Cplsm, Micr	Affected	radixin; RIKEN cDNA 9830163H01 gene	RDX	10585301	−2.012
Neur, Cskel, Cplsm	Decreased	Rho-associated coiled-coil containing protein kinase 2	ROCK2	10394731	1.920
Neur	Decreased	sparc/osteonectin, cwcv and kazal-like domains proteoglycan 2	SPOCK2	10363415	1.554
Cskel, Cplsm, Micr	Decreased	serine/threonine kinase 11	STK11	10364683	−1.902
Cskel, Cplsm	Affected	STE20-related kinase adaptor beta	STRADB	10346576	1.515
Neur	Decreased	synaptic Ras GTPase activating protein 1 homolog (rat)	SYNGAP1	10443108	1.762
Cskel, Cplsm, Micr	Decreased	transforming growth factor, beta 3	TGFB3	10401673	−1.655
Cskel, Cplsm, Micr	Decreased	tenascin R	TNR	10350951	1.645
Neur	Decreased	tuberous sclerosis 2	TSC2	10448631	1.801
Cskel, Cplsm, Micr	Decreased	ubiquitination factor E4B, UFD2 homolog (S. cerevisiae)	UBE4B	10518642	1.732
Cplsm	Affected	unconventional SNARE in the ER 1 homolog (S. cerevisiae)	USE1	10572580	−1.758
Neur, Cskel, Cplsm, Micr	Increased	vimentin	VIM	10469322	−2.380

* Neur: growth of neurites; Cskel: organization of cytoskeleton; Cplsm: organization of cytoplasm; Micr: microtubule dynamics.

** Increased: gene expression pattern predicts an increase in the specific function; Decreased: gene expression pattern predicts a decrease in the specific function; Affected: gene is involved in specific function, but unclear how it would influence it.

## Discussion

Neurons are particularly vulnerable to the consequences of viral infection and/or the anti-viral immune response in the brain. Evidence suggests that neuropathology induced directly or indirectly by infection may lead to symptoms of disease. For example, caspase-dependent apoptosis has been implicated in pathogenesis of vesicular stomatitis virus (VSV) and WNV, and cytomegaloviruses induce lysis of the host cell [37]–[39]. For RABV, the basis of neuropathogenesis is unknown, and predicted to be due either to neuronal death or dysfunction.

A challenge in studying the longevity of infected neurons is identifying and isolating them after resolution of the acute viral infection. To study rabies, approaches used in the past have included histopathology, measurements of cell death (via TUNEL assay), and *in vitro* studies that may not represent the biology of an infected neuron *in vivo*. These assays have known limitations and may account for the inconsistent results in this research area, making it hard to draw a conclusion as to neuronal cell fate after viral infection. Here we examined the survival and functionality of neurons infected with RABV using a novel and innovative approach: the Cre reporter mouse model. Of note, our results are based on attenuated RABV which only models what may happen in a natural RABV infection. We appreciate the fact that pathogenic rabies virus is invariably lethal and few survive to the later timepoints evaluated in this study. However, by using a less virulent strain of virus, we were able to sufficiently infect brain neurons without killing the mice, and study the impact of the infection on these cells long after clearance of substantial inflammation and bystander effects, which may complicate data analysis. More pathogenic RABV variants could be used, though the window of time between infection and death is brief and catching these cells for analysis would be challenging. However, we speculate that in the rare event of an animal (or person) surviving a RABV infection, the changes within the previous infected neurons are similar to what have been observed for the attenuated RABV. Though we do not have direct evidence that infection with a pathogenic RABV will result in similar changes in gene expression as observed here for the attenuated RABV, there is indirect support for this hypothesis. This is based on the findings that the major difference between a pathogenic and attenuated RABV is the faster spread within the CNS of the pathogenic strain [40] which, in combination with the lack of immunogenicity of the pathogenic strain, normally results in death of the infected host. However, the lack of any pathology within the RABV-infected brain, which is especially true for an infection with pathogenic RABV strain, [15], [41]–[45] strongly argues for functional changes within the infected neurons, including pathogenic strains.

Our results show that experimental RABV does not induce cell loss as a result of direct or indirect cytopathic effects of the virus, nor a cytotoxic immune response. RABV G has been implicated in apoptotic signaling *in vitro*; for many years it was believed that the quantity of RABV G expressed correlated with both apoptosis and pathogenicity [15], [46]. Recently this paradigm was challenged, and abundance alone was shown not to be the only determinant of apoptosis [47]. Préhaud et al. demonstrated that pathogenic and attenuated strains recruit different intracellular proteins that mediate either cell survival or cell death, respectively [18]. The *in vivo* relevance of their work remains to be determined; *in vitro* infection of pure cultures of neurons, at a high multiplicity of infection, in the absence of an intact immune response may not represent events *in vivo*. Our results indirectly support the claims that RABV immunity is rapid and mediated by non-cytolytic immune responses, likely antibody and cytokine secretion [4]. The role of type I interferons, specifically, has been of great research interest. RABV-infected neurons can produce type I interferons *in vitro* [48], and interferon-α receptor knock out mice are unable to control the virus and ultimately succumb to infection [49]. What impact these cytokines have on neuronal integrity is unclear. Though CD8+ T cells have been found in brain sections of human rabies victims [50], their deletion appears to have no significant effect on the survival of challenged mice [4].

Our data supports the hypothesis that RABV pathogenesis is not due to loss of neurons, but rather to neuronal dysfunction. Evolutionarily this makes sense, as preservation of the neuronal network by inhibition of apoptosis and limitation of inflammation is advantageous for RABV to complete its lifecycle. The Cre reporter model allowed us to isolate “cured” neurons from mice 3 months post-infection to study their transcriptional profiles compared to uninfected cells from the same mice. Comparing one cell population to another in the same mouse acted as an internal control, avoiding differences based on age, treatment, and normal biological variance. We show evidence that neurons exhibit permanent transcriptional differences from uninfected cells long after resolution of the viral infection. We used a bioinformatics tool, Ingenuity Pathway Analysis (IPA), to identify biological functions most significantly impacted by this dysregulated gene set. Our results showed a modest change in gene transcription (rarely over 3-fold difference between infected and uninfected) with most affected genes involved in cell-to-cell signaling and nervous system development/function. The most interesting aspect of the transcriptional data was the prediction of functional consequences potentially induced by the pattern of gene changes: decreased neurite growth, decreased organization of cytoskeleton, decreased organization of cytoplasm, and decreased microtubule dynamics. For example, we observed a down-regulation of many key microtubule/microtubule-associated genes by microarray. Our results, which were acquired through an unbiased assay, are in agreement with others; Li et al. found similar down-regulation of microtubule-related genes in acute pathogenic RABV infection, and this directly correlated to degeneration of neuronal processes [51]. Furthermore, decreased neurite growth and dendritic/axonal swelling has been detected by microscopic examination of infected neurons in culture [11], [12], [18], [51], though it may be strain dependent. Together, these hypothesized gene expression changes may reflect an overall failure to reorganize the cytoskeleton for growth or repair of cellular processes. Future examination of live EGFP labeled cells purified from the brains of these Cre reporter mice may fully elucidate the functionality, or lack thereof, of once-infected neurons compared to never-infected neurons. Furthermore, alternative approaches, such as laser capture microdissection, may be useful in future efforts to monitor transcriptional changes in specific sub-populations of neurons.

We anticipate that an understanding of the deficiencies of RABV infected cells will provide useful insights into the development of novel treatment options for the acute disease based on restoring lost cellular function. The incubation period, or period between infection and development of symptoms, is extremely variable and can range from 1 week to over 1 year in duration. Post-exposure prophylaxis is highly effective during this period; however, once symptoms develop, survival is extremely rare. Our data provides hope that living neurons may be a possible target for intervention. If research can elucidate the precise effect of viral infection of brain neurons, it may be possible to develop novel treatments effective during these late stages of disease. This data also provides reason to be concerned with the use of recombinant rabies vectors for treatment of neurological disorders or infections.

This model may also be used as a tool to study the regenerative capacity of neurons. We looked at neuronal gene expression from one time point only, but analyzing the expression of select genes at several points over a period of time would provide information regarding the ability of these cells to regenerate. Furthermore, viral genome was still present at the 3 month timepoint; is functionality regained after complete genome clearance? Latent herpesvirus induces neuronal deficits, though this is likely due to transcription of latency-associated genes [52]. We believe that the RABV genome is persisting due to the stabilizing effects of the viral nucleocapsid as described for other negative-stranded RNA viruses, including VSV [53], and RABV does not express latency-associated genes. We do not anticipate that the persistent genome will play a role in neuronal dysfunction, though this would need to be confirmed experimentally.

Lastly, this model may be used to study the link between viral infection and chronic neurodegenerative diseases. This link has been suggestive for other abortive or latent viral infections in which the host survives (e.g. herpesviruses, measles, Polio, Epstein-Barr). Multiple sclerosis, for example, is suspected by some of having a viral etiology. A virus-induced disturbance may be, in a subset of patients, the starting point for autoimmune demyelination. There is compelling evidence for involvement of *Human herpesvirus 6* (HHV-6), as viral antigen has been found in sclerotic lesions, and virus-specific IgM antibodies increase in relapsing-remitting MS patients [54], though proving a causative link between viral infection and chronic dysfunction has been difficult. The Cre mouse model proposed here provides a powerful tool to study other neurotropic viruses and show causation between virus and chronic disease. Our data clearly demonstrates that neurotropic viral infection can cause persistent neurological deficits long after active infection has been cleared, and that once-infected neurons may not be restored to their pre-infection phenotype. The implications of these findings are substantial, as surviving neurons, which have a remarkable capacity to recover following damage [55], [56], may be targeted for novel treatment options in rabies and other neurological infections by restoring compromised cellular functions.

## Materials and Methods

### Mice

All animals were handled in strict accordance with good animal practice as defined by the relevant international (Association for Assessment and Accreditation of Laboratory Animal Care (AAALAC) (Accreditation Status TJU: Full)) and national (TJU Animal Welfare Assurance Number: A3085-01), and all animal work was approved by the Institutional Animal Care and Use Committee (IACUC) at Thomas Jefferson University TJU. Animal use protocols are written and approved in accordance with Public Health Service Policy on Humane Care and Use of Laboratory Animals, The Guide for the Care and Use of Laboratory Animals. TJU IACUC protocol number 414 I (Pathogenesis of rabies virus and vaccine vectors in mice) was utilized in this study. Cre reporter mice, strain B6.129(Cg)-*Gt(ROSA)26Sor^tm4(ACTB-tdTomato,-EGFP)Luo^*/J, were purchased from the Jackson Laboratory, USA. Heterozygotes were bred and offspring were genotyped as recommended by Jackson Laboratories.

### Mouse infection

Mice were anesthetized with 4% isoflurane by inhalation and inoculated intranasally with 1×10^5^ ffu virus diluted to 20 µL with phosphate-buffered saline (PBS)(10 µL per nostril). Mice were sacrificed at the indicated time points post-infection and brain tissue harvested for RNA and immunohistochemistry.

### Construction, recovery, and characterization of Cre-expressing RABV

cBNSP is an infectious clone based on the RABV vaccine strain SAD B19, which contains two single restriction sites (BsiWI and NheI) for inserting foreign genes [30]. Enterobacteria phage P1 Cre Recombinase (Genbank accession number X03453) was engineered to include a 5′ nuclear localization signal (ATG GCA CCC AAG AAG AAG AGG AAG) to promote high efficiency *in vivo* recombination between *loxP* sites, and is hereafter referred to simply as Cre (Cre cDNA was kindly provided by Dr. Jianke Zhang, Thomas Jefferson University, Philadelphia). The Cre gene was amplified using forward primer 5′-cac CGT ACG acc atg gca ccc aag aag aag-3′ (BsiWI site in caps, ATG start codon for the nuclear localization signal is underlined) and reverse primer 5′-cga GCT AGC cta atc gcc atc ttc cag cag g-3′ (NheI site in caps), and cloned into cBNSP using the unique BsiWI and NheI sites between RABV nucleoprotein (N) and phosphoprotein (P). The resulting cDNA was termed RABV-Cre. RABV-Cre was recovered as previously described [30].

Multi- and single-step growth curves were conducted on BSR cells as previously described [29]. Cre functionality was evaluated *in vitro* by infecting primary fibroblasts cultured from Cre reporter mice. To isolate primary fibroblasts, leg muscle was dissected from euthanized adult Cre reporter mice and dissociated using a protocol adapted from Blau et al. [57]. Briefly, muscle tissue was minced into 3- to 4-mm pieces in PBS, incubated in an enzyme mixture of collagenase D (0.75 units/mL, Roche Applied Science, 11088858001), Dispase II (1.2 units/mL, Roche Applied Science, 10295825001), and 2.5 mM CaCl_2_ for 45 min in a 37°C water bath, triturating with a 5 mL pipette 3 times throughout the incubation. Ten milliliters of PBS was added to the cell suspension and passed through a 70 µm filter, spun at 300×*g* for 5 min, resuspended in DMEM supplemented with 10% FBS and penicillin-streptomycin (DMEM10), and plated in a standard T75 tissue culture flask. To enrich for fibroblasts, cells were allowed to attach to the flask for 1 h at 37°C, at which time the unattached cells were aspirated and the adherent cells were passaged in DMEM10 for 5 to 7 d. Cre reporter primary fibroblasts plated at 80–90% confluency in 6-well plates were infected with 2.4×10^6^ ffu recombinant virus at 37°C. After 96 h, the cells were assayed for color change from TdTomato to EGFP by fluorescent imaging and flow cytometry. For imaging, plated cells were fixed with 4% PFA (pH 7) for 20 min at 4°C and viewed under a fluorescence microscope. For flow cytometry, trypsinized cells were fixed in suspension with 4% PFA (pH7) for 20 min at 4°C, washed once in PBS supplemented with 2% BSA, and analyzed on a BD FACSCalibur (50,000 events collected).

### Immunoblotting

NA cells plated at 80–90% confluency in 12-well plates were infected with 3×10^6^ ffu recombinant virus at 37°C in serum-free media. After 1 h, inoculum was replaced with RPMI supplemented with 5% FBS and penicillin-streptomycin and incubation was continued at 34°C. After 48 h, the cells were washed in PBS, lysed on ice in RIPA buffer (25 mM Tris pH 7.5, 150 mM NaCl, 1% NP-40, 0.4% sodium deoxycholate, 1 mM EDTA) containing 1× protease inhibitor cocktail (Sigma), and centrifuged at 12,000×*g* for 10 min. The protein concentration in the supernatant was determined using a BCA kit (Pierce, 23227) and the supernatants were denatured with urea buffer (125 mM Tris–HCl pH 6.8, 8 M urea, 4% sodium dodecyl sulfate, 5% beta-mercaptoethanol, 0.02% bromophenol blue) at 56°C. Five micrograms of protein were resolved on an SDS-10% polyacrylamide gel and transferred to a nitrocellulose membrane in Towbin buffer (192 mM glycine, 25 mm Tris, 20% methanol). The membrane was then blocked in TBST (100 mM Tris-HCl pH7.9, 150 mM NaCl, 0.01% Tween20) containing 5% dried milk at 4°C for several hours. After blocking, the membrane was incubated overnight with rabbit anti-Cre polyclonal antibody (Novagen, 69050) at a dilution of 1∶10,000, anti-RABV serum from reconvalescent RABV-infected mice diluted 1∶6,000, and a mouse monoclonal antibody against Actin (Sigma, A5441), all diluted 1∶250,000 in TBST containing 5% BSA. After washing, the blot was incubated for 1 h in anti-rabbit-HRP conjugate and anti-mouse-HRP conjugate, both diluted 1∶50,000 in blocking buffer. Bands were developed with SuperSignal West Pico Chemiluminescent substrate (Pierce, 34080).

### Tissue harvest

Immediately after dissection, the mouse brains were bisected laterally using a sterile scalpel. One half was immediately immersed in RNAlater (Qiagen, 1 mL/100 mg tissue) for the purpose of RNA isolation. The second half was placed in 4% PFA (pH 7.0) for immunohistochemical analysis.

### Immunohistochemistry

Immediately after dissection, brains were fixed 24 h in 4% paraformaldehyde (pH 7.0) and cryoprotected by sequential saturations in 10%, 20%, and 30% sucrose/PBS (each for 24 h). Samples were embedded, frozen, and cut by the Kimmel Cancer Center's Pathology Core Facility. Brains were embedded in Tissue-Tek O.C.T. compound (Sakura), frozen and cut at −20°C on a Microm HM550 cryostat (Thermo Scientific) into 10 µm sections, and mounted onto charged slides (Thermo Scientific Superfrost Plus). Slides were stored at −20°C and either directly imaged or stained for cell- and virus-specific antigens and then imaged. In preparation for staining, sections were permeabilized in 0.2% TritonX-100/PBS for 1 h at room temperature (RT), washed in 0.05% TritonX-100/PBS (wash buffer), and blocked in wash buffer supplemented with 5% BSA. For neuronal staining, slides were stained with a 1∶100 dilution of mouse anti-NeuN (MAB377; Millipore), washed 3×, stained with 1∶300 dilution of Pacific Blue goat anti-mouse (P-10993; Invitrogen). For astrocyte staining, slides were stained with a 1∶250 dilution of rabbit anti-GFAP (NB300-141; Novus), washed 3×, stained with 1∶300 dilution of Pacific Blue goat anti-rabbit (P-10994; Invitrogen). For RABV P staining, slides were directly stained with a 1∶300 dilution of AlexaFluor647 mouse anti-RABV P antibody. This antibody was generated by conjugating AF647 (A20173; Invitrogen) to purified RABV P-specific IgG produced from hybridoma cells kindly provided by Dr. Danielle Blondel, Gif sur Yvette, France [58]. Specifically, antibody-containing supernatant was purified using Nunc ProPur Midi G Kit, dialyzed in PBS, and conjugated. Images were acquired using a Leica DM5000B fluorescence microscope equipped with the DFC340FX camera or a Zeiss LSM 510 META Confocal microscope.

### RNA isolation

Brain tissues immersed in RNAlater were transferred to RLT Buffer (Qiagen) supplemented with beta-mercaptoethanol (10 µL BME/1 mL RLT) at a ratio of 100 µL RLT-BME per 10 mg tissue. Tissue was homogenized with Hard Tissue Omni Tip probes (Omni International). RNA was isolated using the RNAeasy Mini Kit (Qiagen) according to the manufacturer's protocol. A 15 min on-column DNaseI digest (Qiagen) was included for all samples during the purification. RNA concentration and purity were determined using the NanoDrop 2000c (Thermo Scientific).

### Reverse transcription and real-time quantitative PCR

RNA was reverse transcribed into cDNA using the Omniscript Reverse Transcription Kit (Qiagen) according to the manufacturer's protocol. Each 20 µL reaction contained 2 µg of purified RNA, 10 units RNaseOut ribonuclease inhibitor (Invitrogen), and 0.5 µM primer. Reaction mixtures were incubated at 37°C for 1 h, followed by 5 min at 95°C to inactivate the enzyme. All primers/probes used throughout were designed using Sigma-Aldrich's OligoArchitect and purchased from Sigma-Genosys. The following reverse transcription primers were used, 5′ to 3′: RV-N genome (CAT GGA ACT GAC AAG AGA), messenger RNA (for subsequent QPCR of RV-N sense message; TTT TTT TTT TTT TTT TTT TTV; V = G, C, or A), EGFP (CGG ATC TTG AAG TTC ACC), RPL13A housekeeping gene (CTT TTC TGC CTG TTT CCG TA; part of MHK-1 primer set from RealTimePrimers.com). Quantitative analysis of all genes was conducted on the MX3005P QPCR Machine (Agilent Technologies). All genes (except RPL13A, described below) were quantified using the QuantiFast Probe PCR Kit containing ROX internal reference dye (Qiagen, 204256) according to the manufacturer's protocol. Each of these 20 µL reactions contained 2 µL of the reverse transcription reaction, 0.4 µM each primer, and 0.2 µM TaqMan probe. QPCR cycling began with one hot start cycle of 95°C for 15 min, followed by 45 amplification cycles of 95°C for 15 sec, and 60°C for 1 min (data acquired at end of each step). RPL13A was quantified using Brilliant II SYBR Green QPCR Master Mix (Agilent; 600828) according to the manufacturer's protocol. Each of these 20 µL reactions contained 2 µL of the reverse transcription reaction and 0.1 µM each primer. QPCR cycling began with one cycle of 95°C for 15 min, followed by 45 cycles of 95°C for 30 sec, 58°C for 1 min (data acquired at end of this step), and 72°C for 30 sec. Sequences of the QPCR forward primer, reverse primer, and TaqMan probe (respectively) are as follows, 5′ to 3′: RV-N anti-sense genome and RV-N sense message (CAT GGA ACT GAC AAG AGA, TGC TCA ACC TAT ACA GAC, [6-FAM]ATG CGT CCT TAG TCG GTC TTC TC[TAMRA]), EGFP (AGC TGG AGT ACA ACT ACA, CGG ATC TTG AAG TTC ACC, [6-FAM]ATG CCG TTC TTC TGC TTG TCG[TAMRA]), RPL13A housekeeping gene (ATG ACA AGA AAA AGC GGA TG, CTT TTC TGC CTG TTT CCG TA, [no probe]; primers from MHK-1 primer set from RealTimePrimers.com). Primer pairs for all genes were validated by measuring product linearity (R^2^>0.99) and amplification efficiency (E = 98–102%). SYBR Green reactions were further validated for specificity by running dissociation curves after quantification (using default program on MX3005P) and by running PCR products on 2.5% agarose gel. All samples were run in triplicate alongside negative controls (water and No RT). For absolute quantification of RV-N anti-sense genome and RV-N sense message, an eight point standard curve was generated from 10-fold serial dilutions of cDNA of known copy number (ranging 10^8^ to 10^1^ transcripts). Copy numbers were normalized to RPL13A housekeeping gene. For relative quantification of EGFP, the DDCt method [59] was used to measure fold-change of EGFP relative to RPL13A housekeeping gene.

### Brain tissue dissociation and EGFP^+^ FACS purification

Whole brains with olfactory bulbs attached were dissected from adult (>8 wks) Cre reporter mice and dissociated using a well-described protocol adapted from Huettner and Baughman [60], [61]. Brain tissue was minced into 3- to 4-mm pieces in Earle's Balanced Salt Solution (EBSS, HyClone, SH3002902). Papain enzyme (Worthington Biochemical, LS003126) was preactivated in EBSS/0.5 mM EDTA/1 mM L-cysteine by incubating 10 min at 37°C. Minced tissue was incubated in 20 units/mL preactivated papain and 125 units/mL DNase I (Worthington Biochemical, LS002006) for 85 min in a 37°C water bath, triturating gently with a 10 mL pipette twice throughout the incubation. The cell suspension was passed through a 70 µm filter, spun at 300×*g* for 5 min, and resuspended in 3 mL of dilute protease inhibitor solution (EBSS, 1 mg/mL ovomucoid inhibitor [Worthington Biochemical, LS003085], 1 mg/mL BSA, 125 units/mL DNase I). A discontinuous density gradient was made by layering the cell suspension on top of 5 mL concentrated protease inhibitor (EBSS, 10 mg/mL ovomucoid inhibitor, 10 mg/mL BSA) and centrifuging at 70×*g* for 8 min to separate cells (pellet) from debris (supernatant). Cells were washed once in PBS supplemented with 2% BSA, and FACS-purified based on EGFP and TdTomato fluorescence utilizing a Coulter MoFlo sorter. EGFP-positive and EGFP-negative populations were saved for RNA extraction.

### RNA purification and cDNA amplification

RNA was isolated using the RNAeasy Mini Kit (Qiagen) according to the manufacturer's protocol. RNA was quantified on a NanoDrop 2000c (Thermo Scientific), followed by RNA quality assessment on an Agilent 2100 Bioanalyzer (Agilent, Palo Alto, CA, USA). Amplification and labeling was performed using the Ovation Pico WTA-system V2 RNA amplification system (NuGen Technologies, Inc.). Briefly, 50 ng of total RNA was reverse transcribed using a chimeric cDNA/mRNA primer, and a second complementary cDNA strand was synthesized. Purified cDNA was then amplified with ribo-SPIA enzyme and SPIA DNA/RNA primers (NuGEN Technologies, Inc.). Amplified DNA was purified with Qiagen MinElute reaction cleanup kit. The concentration of Purified ST-cDNA was measured using the Nanodrop. 2.5 µg ST-cDNAs were fragmented and chemically labeled with biotin to generate biotinylated ST-cDNA using FL-Ovation cDNA biotin module V2 (NuGen Technologies, Inc.).

### Microarray

Affymetrix gene chips, mouse gene 1.0 ST array (Affymetrix, Santa Clara, CA), were hybridized with fragmented and biotin-labeled target (2.5 µg) in 110 µl of hybridization cocktail. Target denaturation was performed at 99°C for 2 min and then 45°C for 5 min, followed by hybridization for 18 h. Arrays were then washed and stained using Genechip Fluidic Station 450, and hybridization signals were amplified using antibody amplification with goat IgG (Sigma-Aldrich) and anti-streptavidin biotinylated antibody (Vector Laboratories, Burlingame, CA, USA). Chips were scanned on an Affymetrix Gene Chip Scanner 3000, using Command Console Software. Background correction and normalization were done using Iterative Plier 16 with GeneSpring V11.5 software (Agilent, Palo Alto, CA, USA). 1.5-fold differentially expressed gene list was generated. The differentially expressed gene list was loaded into Ingenuity Pathway Analysis (IPA) 5.0 software (http://www.ingenuity.com) to perform biological network and functional analyses. The microarray data can be accessed at the GEO - repository access number GSE38975 (http://www.ncbi.nlm.nih.gov/geo/query/acc.cgi?acc=GSE38975)

## Supporting Information

Figure S1 Fluorescence-activated cell sorting (FACS) of brain cells isolated from infected or naive Cre reporter mice three months post-infection. EGFP+ cells were collected from the “infected” gate and used for gene expression analysis.
(TIFF)

Table S1 **Microarray analysis of RABV-infected neurons 3 months after infection.** Our analysis identified 1248 genes differentially expressed between the sorted groups (infected vs. uninfected, ≥1.5 fold change, p<0.05), 361 genes of which were down-regulated, 887 genes that were up-regulated. Gene expression levels ranged from 3.09-fold up-regulation to 2.72-fold down-regulation.
(XLSX)

Table S2 **Microarray analysis of RABV-infected neurons 3 months after infection.** The table shows “neuron-specific genes” up- or down-regulated in RABV infected Cre-mice.
(XLSX)
