# Supplementary figures and images for: Immune Clearance of Attenuated Rabies Virus Results in Neuronal Survival with Altered Gene Expression
Source: PLoS Pathog. 2012 Oct 11;8(10):e1002971. doi: 10.1371/journal.ppat.1002971 (PMC3469654; doi:10.1371/journal.ppat.1002971)

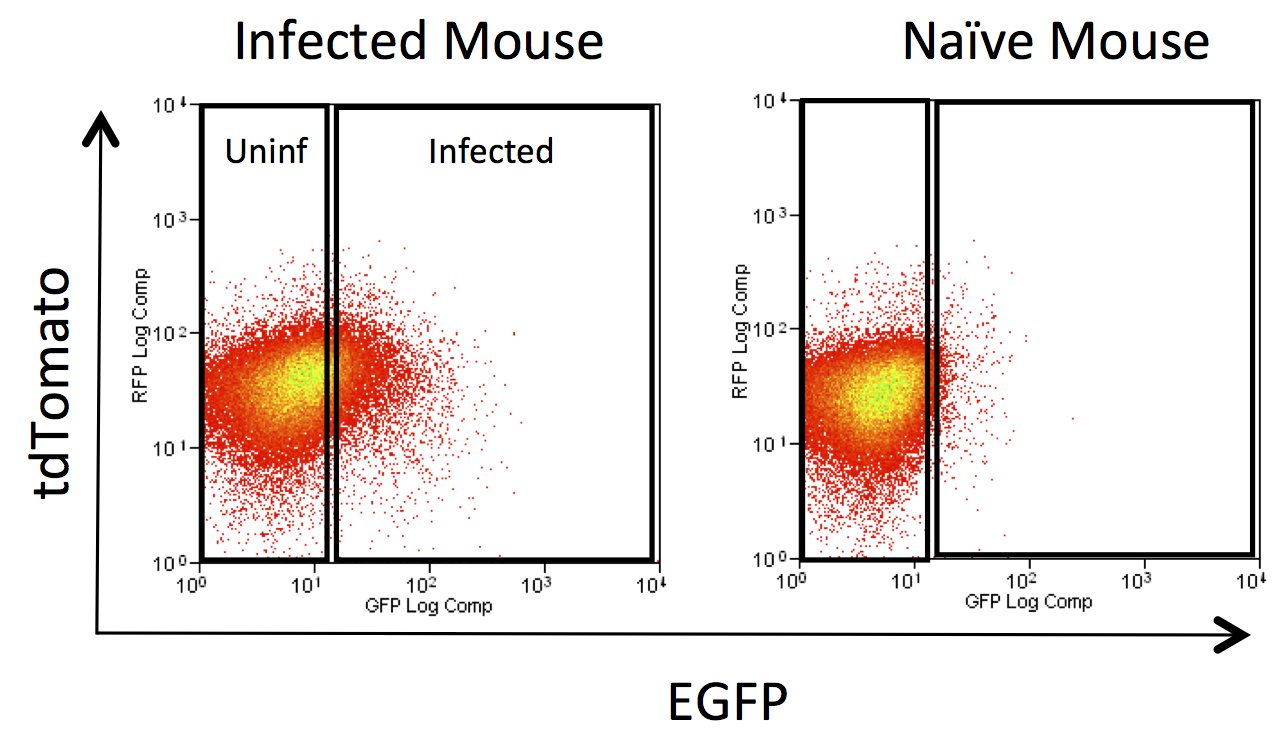

Supplement: Figure S1 — Fluorescence-activated cell sorting (FACS) of brain cells isolated from infected or naive Cre reporter mice three months post-infection. EGFP+ cells were collected from the “infected” gate and used for gene expression analysis. (TIFF) [file ppat.1002971.s001.tiff]
